# 

**Published:** 1886-08

**Authors:** 


### Contents—August, 1886.

ORIGINAL COMMUNICATIONS—Impetigo Contagiosa: Dr. E. J. Beall ........ 47
Colpo-Myotomy: Dr. J. W. Carhart ........ 59
Ligation of the Brachial Artery: Dr. N. B. Kennedy. 63
CORRESPONDENCE—Watermelon Seed in Trachea, Dr. Pickett 66
Religion in Medical Addresses endorsed; the minority report ........ 67
The Presidential Address and Modern Science, Dr. Schmitt ........ 68
SOCIETY NOTES—Bell County Medical Association ........ 70
An International Medical Society in Texas ........ 70
CULLINGS FROM EXCHANGES—Sweet Malaria: Can't understand it ........ 71-72
EDITORIAL—An American Contribution to Bacteriology .... 73
Penny Wisdom again ........ 76
EDITORIAL NOTES—Sundry paragraphs too numerous for this limited space—read them ........ 77
BIBLIOGRAPHY—Book Reviews and Notices ........ 83
ADVERTISERS' EDITORIALS ........ 87

